# Trends in Anaphylaxis Hospitalizations among Adults in Spain and Their Relationship with Asthma—Analysis of Hospital Discharge data from 2016 to 2021

**DOI:** 10.3390/healthcare11233016

**Published:** 2023-11-22

**Authors:** Francisco J. Caballero-Segura, Natividad Cuadrado-Corrales, Rodrigo Jimenez-Garcia, Ana Lopez-de-Andres, David Carabantes-Alarcon, Jose J. Zamorano-Leon, Francisco Carricondo, Barbara Romero-Gomez, Javier De-Miguel-Díez

**Affiliations:** 1 Respiratory Department, Hospital General Universitario Gregorio Marañón, Facultad de Medicina, Universidad Complutense de Madrid, Instituto de Investigación Sanitaria Gregorio Marañón (IiSGM), 28007 Madrid, Spain; fcabal01@ucm.es (F.J.C.-S.); javier.miguel@salud.madrid.org (J.D.-M.-D.); 2 Department of Public Health and Maternal & Child Health, Faculty of Medicine, Universidad Complutense de Madrid, 28040 Madrid, Spain; rodrijim@ucm.es (R.J.-G.); anailo04@ucm.es (A.L.-d.-A.); dcaraban@ucm.es (D.C.-A.); josejzam@ucm.es (J.J.Z.-L.); 3 Department of Immunology, Laboratory of Neurobiology of Hearing (UCM 910915), Ophthalmology and Otorhinolaryngology, Faculty of Medicine, University Complutense, IdISSC, 28040 Madrid, Spain; fjcarric@ucm.es (F.C.); brgomez@ucm.es (B.R.-G.)

**Keywords:** anaphylaxis, asthma, epidemiology, hospital admissions, Spain

## Abstract

(1) Background: Anaphylaxis is a rapid-onset, life-threatening hypersensitivity reaction. This study explores the epidemiological trends and clinical outcomes of adult patients with and without asthma hospitalized for anaphylaxis in Spain from 2016 to 2021. (2) Methods: Data from the Spanish National Hospital Discharge Database (RAE-CMBD) were analyzed. We stratified patients with anaphylaxis based on their asthma diagnosis and evaluated various comorbidities and clinical outcomes. Propensity score matching was used to match confounders. (3) Results: The total number of hospitalizations for anaphylaxis remained stable, with a decrease in 2020 probably due to the COVID-19 pandemic. Drug-induced anaphylaxis increased, in addition to being the main triggering factor. Asthma prevalence among those admitted for anaphylaxis emerged from 7.63% to 10.69%, with a higher frequency of respiratory failure and need for mechanical ventilation in this group; despite this, ICU admissions and in-hospital mortality did not differ significantly between asthmatics and non-asthmatics. Asthma was also not a risk factor for severe anaphylaxis. Multivariable analysis identified advanced age, ischemic heart disease, acute respiratory failure, and invasive mechanical ventilation as factors associated with severe anaphylaxis. (4) Conclusions: This study provides valuable information on the complexity of anaphylaxis, its relationship with asthma, and factors influencing its severity. Overall, clinical outcomes did not differ significantly in asthmatic patients compared to non-asthmatic patients, although asthmatic patients had more respiratory complications. Further research is necessary to delve deeper into the multifactorial nature of anaphylaxis and its implications in clinical practice.

## 1. Introduction

Anaphylaxis is a systemic hypersensitivity reaction that is rapid in onset and often life-threatening. This condition typically presents with cutaneous or mucosal changes and may affect the respiratory or circulatory systems [1]. The risk of anaphylaxis has been correlated with bronchial asthma, particularly when food allergies coexist [2]. 

Asthma is a significant health problem in our environment, being a relevant cause of hospital admissions and even mortality. It is a chronic respiratory disease characterized by recurrent airway inflammation, bronchial hyperreactivity, and airflow obstruction [3,4].

Anaphylaxis and asthma are both potentially fatal hypersensitivity reactions. They can coexist, imitate, or exacerbate each other. The cause of their association remains uncertain. However, studies suggest inadequate asthma management is associated with more severe anaphylactic reactions in individuals of all ages. Additionally, in children, asthma is linked to the severity and recurrence of anaphylactic reactions [5]. 

Previous studies have found a correlation between asthma and anaphylaxis, with anaphylaxis risk being 3.3 times higher in people with severe asthma and twice as high in non-severe asthmatics compared to patients without asthma [6]. Distinguishing an asthma exacerbation from anaphylaxis can often be challenging, and in some cases, only the associated extra-respiratory symptoms can help differentiate between the two conditions [7].

Few studies have evaluated the clinical characteristics, comorbidities, and hospital outcomes of patients hospitalized with anaphylaxis who also have asthma, especially in Spain. The main objective of our study was to evaluate the temporal trend in the incidence, clinical characteristics, comorbidities, and hospital outcomes among patients with and without asthma who had a hospital admission diagnosis of anaphylaxis in Spain from 2016 to 2021. Other objectives were to compare the study variables in patients with and without asthma using propensity score matching (PSM), to identify the variables associated with severe anaphylaxis defined as causing in-hospital mortality (IHM) and/or admission to the intensive care unit (ICU) among patients with asthma, and to evaluate the effect of asthma on the occurrence of severe anaphylaxis.

## 2. Materials and Methods

We conducted an observational, population-based, retrospective study using the Spanish National Hospital Discharge Database (RAE-CMBD, Registro de Actividad de Atención Especializada-Conjunto Mínimo Básico de Datos) as the database. The study period spanned from 1 January 2016 to 31 December 2021.

The RAE-CMBD collects individual patient information (sex, age, admission, and discharge dates), diagnoses (up to 20), procedures (up to 20), and discharge destination (home, voluntary discharge, social institution, and deceased). Diagnoses include conditions present at admission or diagnosed during hospitalization. Procedures include therapeutic or diagnostic procedures performed during admission [8].

The RAE-CMBD uses the International Classification of Diseases, Tenth Revision (ICD-10). All codes used in this research are indicated in Table S1.

The study population included adult patients (18 years or older) with an anaphylaxis code according to ICD-10 in any diagnostic position in the RAE-CMBD database. Subsequently, this population was stratified based on whether they had a code for asthma in any diagnostic position in the database.

The reason for the anaphylactic reaction (food-related, serum-related, drug-related, or unspecified) was recorded. Smoking status and comorbidities were also recorded, including obesity, obstructive sleep apnea, gastroesophageal reflux disease (GERD), chronic rhinitis, atopic dermatitis, anxiety, depression, chronic obstructive pulmonary disease (COPD), hypertension, ischemic heart disease, atrial fibrillation, hypothyroidism, hyperthyroidism, and diabetes mellitus (see codes in Table S1).

Among the specific symptoms and signs of anaphylaxis, the following were recorded: hypotension, syncope/collapse, nausea/vomiting, abdominal pain, acute respiratory failure, and urticaria when they appeared in any diagnostic position in the database (see codes in Table S1).

The use of mechanical ventilation (invasive and non-invasive) was evaluated regardless of its position in the procedures section of the database (Table S1).

Only those patients who stayed for at least 24 h in an intensive care unit (ICU) had a code recorded for this condition. If a patient died during the hospitalization and/or had to be admitted to the ICU, they were classified as suffering “severe anaphylaxis” for study purposes.

### 2.1. Propensity Score Matching

The PSM method is used to control the effect of confounding variables in groups with a significantly different baseline distribution of variables that may be associated with the study outcome [9]. For each patient with asthma, a propensity score (PS) was calculated using multivariate logistic regression and was matched afterwards with a subject without asthma with an equal or very similar PS. We matched 1:1 using calipers, for which the value of the standard deviation (SD) of the logit of the PS had to be ≤0.2 [9]. To assess the goodness of fit of the PSM, love plots are provided showing the absolute standardized differences before and after matching [9].

### 2.2. Statistical Analysis

To describe the study populations, we calculated absolute and relative frequencies and mean with standard deviations for categorical and continuous variables, respectively.

To assess possible changes from 2016 to 2021 in the distribution of study variables, we applied the linear regression t-tests and the Cochran–Mantel–Haenszel statistic.

The Fisher exact test was used to compare proportions and the t-test or Mann–Whitney test to compare means.

Using severe anaphylaxis as the dependent variable, we constructed multivariable logistic regression models to assess the association of study variables with a worse evolution of anaphylaxis. Three models were created: (i) for people with asthma; (ii) for people without asthma; and (iii) for the entire study population. Univariate analysis was used to identify the variables included in the multivariable models. Adjusted odds ratios (OR) along with their 95% confidence intervals (CI) are provided.

As a sensitivity analysis, we repeated the multivariable models, including only those clinical conditions that were present on admission and could not be related to the anaphylactic reaction. We also excluded the mechanical ventilation in this model.

Stata version 14 was the statistical software used.

### 2.3. Ethics Statement

The Spanish Ministry of Health (SMH) is the authority that provides, free of charge, the RAE-CMBD database to any investigator upon request [10]. Our research was done using totally ammonized data that was provided to us by the SMH after an ethical evaluation of our investigation protocol. Therefore, and according to the Spanish law as this administrative registry is mandatory and totally ammonize, no written consent from patients nor approval by an ethics committee are necessary.

## 3. Results

Between 2016 and 2021, there were 5,902 hospital admissions with a code for anaphylaxis in Spain. Of these admissions, 50.27% occurred in women, with a mean age of 60.23 years. Allergy reactions due to drug consumption accounted for 58.76% of anaphylactic reactions, followed by reactions due to food (12.66%) and serum (2.76%). Unspecified reactions accounted for 25.58%.

The presence of an asthma code among those diagnosed with anaphylaxis represented 9.34% (n = 551) of hospital admissions.

Among those diagnosed with anaphylaxis, 31.87% were admitted to the ICU, and the in-hospital mortality rate was 4.78%. The proportion of subjects with anaphylaxis considered severe, defined as being admitted to the ICU and/or dying during hospitalization, was 33.62%.

### 3.1. Temporal Trends in Hospital Admissions for Anaphylaxis in Spain between 2016 and 2021

As shown in Table 1, the number of hospital admissions for anaphylaxis in 2016 was 944, and it was 1029 in 2021. The lowest frequency of admissions occurred in 2020 (n = 841).

Throughout the study period, there was a significant increase in drug-related reactions (47.25% in 2016 vs. 65.4% in 2021; *p* < 0.001) and serum-related reactions (3.28% vs. 3.5%; *p* = 0.028). However, reactions classified as unspecified decreased (36.33% vs. 19.44%; *p* < 0.001).

The presence of asthma increased significantly from 2016 (7.63%) to 2021 (10.69%). Likewise, the use of non-invasive mechanical ventilation increased over the study period (1.27% in 2016 vs. 3.11% in 2021; *p* = 0.003) (Table 1).

The prevalence of severe anaphylaxis remained stable from 2016 to 2021, ranging from 31% to 36% (*p* = 0.179).

### 3.2. Characteristics of Hospital Admissions for Anaphylaxis in Spain Based on the Presence of Asthma

The comparison of characteristics between patients with and without asthma who were admitted with anaphylaxis in Spain between 2016 and 2021 is shown in Table 2. Patients diagnosed with anaphylaxis who had asthma were younger than those without asthma (53.34 vs. 60.94 years; *p* < 0.001). Of the admissions with asthma, 65.52% were female, while in the non-asthma group, it was 48.7% (*p* < 0.001), as indicated in Table 2.

Regarding the type of anaphylactic reaction, patients with asthma had a higher proportion of food-related reactions (23.96% vs. 11.49%; *p* < 0.001), but a lower proportion of drug-related reactions (51.72% vs. 59.48%; *p* < 0.001) and serum-related reactions (1.09% vs. 2.93%; *p* = 0.012) compared to patients without asthma.

As indicated in Table 2, patients with asthma had a significantly higher frequency of GERD, chronic rhinitis, atopic dermatitis, and hypothyroidism, but a significantly lower frequency of COPD, hypertension, ischemic heart disease, atrial fibrillation, and diabetes mellitus compared to patients without asthma.

Regarding specific signs and symptoms of anaphylaxis, patients with asthma had a significantly higher frequency of acute respiratory failure (17.06%) than those without asthma (9.89%).

The use of mechanical ventilation, both invasive and non-invasive, was significantly higher in patients with asthma compared to those without asthma (15.25% and 3.99% vs. 11.08% and 2.47%, respectively).

No differences were found in the ICU admission rate for patients with and without asthma. Nor were any differences found in IHM or in the rate of severe anaphylaxis in patients in both groups of patients, as shown in Table 2.

After performing PSM based on the adjustment variables, as indicated in Table 3, patients with asthma had a higher frequency of nausea/vomiting than non-asthmatics (1.27% vs. 0%; *p* = 0.008) and a higher frequency of acute respiratory failure (17.06% vs. 8.53%; *p* < 0.001). In addition, patients with asthma underwent invasive mechanical ventilation more frequently than non-asthmatics (15.25% vs. 8.89%; *p* = 0.001).

**Figure 1:**
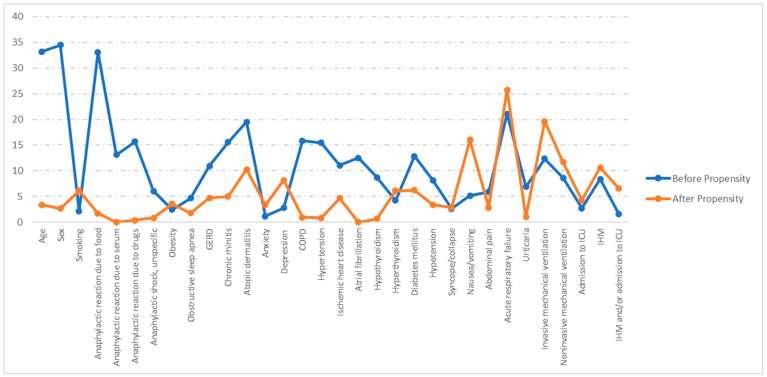
Love plot showing the comparison of covariate values for patients with and without asthma: absolute standardized differences before and after propensity score matching (PSM). GERD: Gastroesophageal reflux disease; COPD: chronic obstructive pulmonary disease. ICU: Intensive care unit. IHM: In-hospital mortality.

Figure 1 shows the absolute standardized differences before and after PSM. A significant imbalance has been ruled out, as all absolute standardized differences after PSM were below 10% [9].

### 3.3. Analysis of Factors Associated with Severe Anaphylaxis during Hospitalization for Anaphylaxis among Patients with Asthma

Table S2 shows the characteristics, chronic conditions, specific signs, and symptoms of hospital admissions with and without a diagnosis of asthma in Spain, 2016–2021, according to the severity of anaphylaxis. In the unavailable analysis, those patients with asthma and higher age, female sex, or having a code for COPD, hypothyroidism, acute respiratory failure, invasive mechanical ventilation, or non-invasive mechanical ventilation had severe anaphylaxis in a significantly higher proportion than those without these characteristics or codes. 

As indicated in Table 4, advanced age (≥65 years) was associated with severe anaphylaxis in the overall study population with anaphylaxis (OR 1.32; 95% CI 1.1–1.58). Additionally, the presence of ischemic heart disease and acute respiratory failure were associated with severe anaphylaxis after multivariable adjustment in those with and without asthma.

Furthermore, undergoing invasive mechanical ventilation in patients with asthma (OR 3.85; 95% CI 1.34–11.05) and in the overall study population (OR 4.21; 95% CI 1.85–9.62) were factors associated with severe anaphylaxis.

Finally, when using the non-asthma status as the reference category, the analysis of the entire database revealed that the presence of asthma was not associated with severe anaphylaxis (OR 0.84; 95% CI 0.63–1.14).

The results of the sensitivity analysis, excluding those variables that could be considered related to anaphylaxis, are shown in Table S3. Among people with asthma, only having a code for ischemic heart disease prior to admission was associated with severe anaphylaxis. In the entire population, age over 64 years and ischemic heart disease were the two variables associated with severe anaphylaxis. As found in the multivariable model with all the covariates, asthma was not associated with severe anaphylaxis. 

## 4. Discussion

The number of patients hospitalized with anaphylaxis remained relatively stable from 2016 to 2021, with 944 cases in 2016 and 1029 in 2021. The most significant decrease occurred in 2020, with 841 instances, coinciding with the COVID-19 pandemic. The exact cause of this decrease remains unclear, but similar trends have been observed in other countries [11,12,13]. 

The most common anaphylactic reactions resulted from drug exposure, and these reactions increased from 2016 to 2021, accounting for 65.4% in the latter year. In contrast, food-related responses remained stable. The upward trend in hospitalizations due to drug-induced anaphylaxis has also been documented in previous studies [14,15,16,17], making it the primary cause of anaphylaxis-related hospitalization in adults [14,18].

Two of the most commonly implicated drug categories are antibiotics and nonsteroidal anti-inflammatory drugs (NSAIDs) [14,15,16]. The rise in cases of drug-induced anaphylaxis may be partly attributed to the aging population and increased polypharmacy [16], especially in the context of intravenous medication [18].

The prevalence of bronchial asthma among anaphylaxis admissions increased from 7.63% to 10.69% between 2016 and 2021. This increase contrasts with the progressive decline in asthma-related hospitalizations observed in multiple countries [19,20].

Among hospitalized patients with anaphylaxis, both in-hospital mortality and the proportion of ICU admissions remained consistent throughout the analyzed period. Mortality due to anaphylaxis showed an increasing trend in Australia, Canada, the United Kingdom, and the United States. This increase was mainly due to cases of drug-induced anaphylaxis. However, the increase in mortality was lower than the overall increase in hospitalizations due to anaphylaxis [21]. A study from Taiwan between 2001 and 2013 clearly illustrates a progressive increase in ICU admissions among patients hospitalized for anaphylaxis [22].

Comparing epidemiological data on hospitalization and mortality due to anaphylaxis among different countries and periods presents methodological challenges. There is still significant heterogeneity in definitions, severity classification, and study design, which hampers the analysis and comparison of results [23].

The percentage of admissions was equal between genders, with around 50% of women in the years under study. However, when considering the concurrent diagnosis of asthma, the percentage of women rises to 65.52%. It is well-documented that women are three times more likely than men to be hospitalized for an asthma-related episode, although the exact cause remains uncertain [24].

Non-asthmatic patients were primarily in the age group over 65 years (46.22%), with only 18.03% falling between the ages of 18 and 44. In contrast, asthmatic patients exhibited a better age distribution throughout the board, with a mean age of 53.34 years, which was lower than that of patients without asthma (60.94 years). In our study, patients with asthma had more reactions due to food (23.96% vs. 11.49%; *p* < 0.001) but fewer reactions due to drugs (51.72% vs. 59.48%; *p* < 0.001) and serum (1.09% vs. 2.93%; *p* = 0.012) than patients without asthma. It’s worth noting that the incidence of food anaphylaxis decreases with age, and it is well-documented that it has the strongest association with bronchial asthma. This factor may partially explain why asthmatic patients were younger than non-asthmatic patients [25,26].

Patients with asthma had a significantly higher frequency of GERD, chronic rhinitis, atopic dermatitis, and hypothyroidism, comorbidities with known linkages to bronchial asthma [27,28,29].

The occurrence of hypertension, ischemic heart disease, atrial fibrillation, and diabetes mellitus was more prevalent in the non-asthmatic population, possibly due to their older age compared to asthma patients. These comorbidities are commonly associated with aging [30].

The incidence of acute respiratory failure was significantly higher among the asthmatic group than the non-asthmatic group (17.06% versus 9.89%). Correspondingly, asthmatic patients had significantly greater usage of mechanical ventilation, including invasive and non-invasive methods, than non-asthmatic patients (15.25% and 3.99% versus 11.08% and 2.47%, respectively).

The occurrence of respiratory failure characterizes severe anaphylaxis [31]. This scenario typically occurs when there is inflammation in the upper airway, bronchospasm, or ventilatory fatigue [32]. Distinguishing between respiratory failure induced by an asthma attack and that caused by anaphylaxis can be challenging in patients with bronchial asthma histories when respiratory symptoms predominate during the anaphylactic reaction. Lack of response to bronchodilator treatment is a factor in differentiating anaphylaxis, along with the many clinical features of anaphylaxis, such as gastrointestinal symptoms or urticaria [33].

It is worth noting that although asthmatic patients face an enhanced likelihood of mechanical ventilation, the overall risk of admission to the ICU and in-hospital mortality did not show significant differences, regardless of the presence of asthma. Various other studies have also observed this phenomenon [34].

In our case, we have not observed an increase in severe anaphylaxis in asthmatic patients compared to non-asthmatic patients. However, it’s important to mention that some other studies suggest that asthma may increase the risk of severe anaphylaxis, albeit with low to moderate evidence [35].

There may be a potential selection bias in hospitalizing patients with anaphylaxis, where patients with asthma may be more likely to be hospitalized even if their anaphylaxis severity is lower, which could partially explain our findings.

After conducting propensity score matching, the majority of disparities between asthmatic and non-asthmatic patients dissolved, affirming the lack of augmented danger for severe anaphylaxis among the asthmatic cohort and the heightened threat of ventilatory failure and mechanical ventilation without a subsequent elevation in inpatient mortality or overall likelihood of ICU admission. There was also no difference between the two groups in terms of the triggering cause of anaphylaxis; drug reactions were the most frequent trigger of hospitalization for anaphylaxis in both groups. 

Severe anaphylaxis is correlated with advanced age (65 years), ischemic heart disease, acute respiratory failure, and the requirement for invasive mechanical ventilation after multivariate adjustment. Previous studies found associations between advanced age, cardiac comorbidity, and drug-induced anaphylaxis and severe anaphylaxis [36,37,38]. 

The study has various strengths and limitations. Concerning its strengths, the study effectively analyzed a substantial sample size derived from the Spanish National Hospital Discharge Database (RAE-CMBD), encompassing patients hospitalized for anaphylaxis nationwide over six years, employing a consistent data analysis method throughout the entire series. The RAE-CMBD collects hospital discharges from most hospitals in Spain, both public and private, extending the Minimum Basic Data Set of Hospital Discharges since its implementation in 2016 [8,39]. In addition, few studies have been able to evaluate the set of patients hospitalized for anaphylaxis according to the presence of asthma in a population of similar size. The use of propensity score matching (PSM) enhances the validity of the findings by reducing the influence of confounding variables.

Regarding limitations, using administrative data from the RAE-CMBD introduces the possibility of classification and coding errors inherent in this data type. These data also lack clinical information that would have been useful, such as asthma severity, degree of control, specific allergens, and pharmacological treatments used. Another relevant limitation is that the study does not allow us to establish causal relationships between variables. Although the research identifies associations, causality remains uncertain, and further research is required to explore the associations found. However, methodological differences in data collection, study designs, heterogeneity in definitions and classifications, and unique epidemiological factors within the Spanish healthcare environment impose limitations on comparing the results obtained in this study with those of others. Nevertheless, it is noteworthy that the health system in Spain maintains almost universal health coverage [40], which, together with the fact that it is a country with a large population, improves the external validity of the results obtained.

Finally, we have used a 1:1 PSM in order to minimize bias, as recommended by Ausitn PC [41]. Furthermore, there are additional limitations to having more than 1:1 matching; in particular, it can make estimation of the variance more difficult, and the risk of increasing the ratio to 1:2 is that incomplete matching occurs when there are no appropriate matched pairs available [41].

## 5. Conclusions

In conclusion, our analysis of anaphylaxis hospitalization data revealed distinct trends. The number of admissions remained relatively stable throughout this period, with a noteworthy decline in 2020. The primary cause of anaphylaxis among hospitalized patients during the analyzed period was drug-induced, and there was a rising trend. The presence of bronchial asthma increased among admissions for anaphylaxis, which contrasts with the downward trend in hospitalizations for asthma as a principal diagnosis observed in multiple countries. Despite the higher frequency of ventilatory failure and mechanical ventilation in asthmatic patients with anaphylaxis, there was no significant difference in ICU admissions or in-hospital mortality between asthmatics and non-asthmatics. Nor did asthma pose a risk of severe anaphylaxis, which contrasts with other studies that do find this correlation, although more markedly so in the pediatric population. Propensity score matching helped with weight confounders, highlighting advanced age, ischemic heart disease, respiratory failure, and the need for invasive mechanical ventilation as factors associated with severe anaphylaxis. 

In light of the heterogeneity of definitions and study designs across countries and periods, meaningful epidemiological comparisons pose a challenge. Nevertheless, this study provides valuable insights into the complex landscape of anaphylaxis, its relationship to asthma, and factors influencing its severity. Further research is required to gain a more comprehensive understanding of the multifaceted nature of anaphylaxis and its clinical implications.

## Figures and Tables

**Table 1:** Characteristics of hospital admissions with a diagnosis of anaphylaxis in Spain, 2016–2021.

Year	2016	2017	2018	2019	2020	2021	*p* Trend
N	944	1002	1057	1029	841	1029	NA
Age, mean (SD)	60.81 (18.09)	59.82 (17.37)	60.52 (17.16)	60.55 (17.88)	59.15 (17.25)	60.37 (18.05)	0.373
18–44 years, n (%)	178 (18.86)	194 (19.36)	197 (18.64)	206 (20.02)	169 (20.1)	212 (20.6)	0.354
45–64 years, n (%)	330 (34.96)	378 (37.72)	378 (35.76)	346 (33.62)	323 (38.41)	347 (33.72)	
≥65 years, n (%)	436 (46.19)	430 (42.91)	482 (45.6)	477 (46.36)	349 (41.5)	470 (45.68)	
Women, n (%)	504 (53.39)	490 (48.9)	517 (48.91)	516 (50.15)	413 (49.11)	527 (51.21)	0.303
Smoking, n (%)	118 (12.5)	171 (17.07)	159 (15.04)	157 (15.26)	126 (14.98)	171 (16.62)	0.086
Anaphylactic reaction due to food, n (%)	120 (12.71)	121 (12.08)	130 (12.3)	137 (13.31)	125 (14.86)	114 (11.08)	0.232
Anaphylactic reaction due to serum, n (%)	31 (3.28)	34 (3.39)	28 (2.65)	14 (1.36)	20 (2.38)	36 (3.5)	0.028
Anaphylactic reaction due to drugs, n (%)	446 (47.25)	483 (48.2)	657 (62.16)	672 (65.31)	537 (63.85)	673 (65.4)	<0.001
Anaphylactic shock, unspecified, n (%)	343 (36.33)	362 (36.13)	245 (23.18)	201 (19.53)	159 (18.91)	200 (19.44)	<0.001
Asthma, n (%)	72 (7.63)	91 (9.08)	89 (8.42)	116 (11.27)	73 (8.68)	110 (10.69)	0.041
Invasive mechanical ventilation, n (%)	85 (9)	110 (10.98)	120 (11.35)	128 (12.44)	103 (12.25)	131 (12.73)	0.110
Non-invasive mechanical ventilation, n (%)	12 (1.27)	15 (1.5)	33 (3.12)	31 (3.01)	31 (3.69)	32 (3.11)	0.003
Admission to ICU, n (%)	291 (30.83)	299 (29.84)	360 (34.06)	342 (33.24)	272 (32.34)	317 (30.81)	0.286
IHM, n (%)	40 (4.24)	46 (4.59)	61 (5.77)	43 (4.18)	50 (5.95)	42 (4.08)	0.192
Severe anaphylaxis (IHM or/and admission to ICU), n (%)	303 (32.1)	317 (31.64)	383 (36.23)	357 (34.69)	291 (34.6)	333 (32.36)	0.179

IHM: In-hospital mortality; ICU: Intensive Care Unit. *p* value for the time trend.

**Table 2:** Characteristics, chronic conditions, specific signs, symptoms, and hospital outcomes of hospital admissions with a diagnosis of anaphylaxis in Spain, 2016–2021, according to the presence of asthma.

	Asthma	No Asthma	*p*
N	551	5351	NA
Age, mean (SD)	53.34 (19.02)	60.94 (17.34)	<0.001
18–44 years, n (%)	191 (34.66)	965 (18.03)	<0.001
45–64 years, n (%)	189 (34.3)	1913 (35.75)	
≥65 years, n (%)	171 (31.03)	2473 (46.22)	
Women, n (%)	361 (65.52)	2606 (48.7)	<0.001
Smoking, n (%)	88 (15.97)	814 (15.21)	0.637
Anaphylactic reaction due to food, n (%)	132 (23.96)	615 (11.49)	<0.001
Anaphylactic reaction due to serum, n (%)	6 (1.09)	157 (2.93)	0.012
Anaphylactic reaction due to drugs, n (%)	285 (51.72)	3183 (59.48)	<0.001
Anaphylactic shock, unspecified, n (%)	128 (23.23)	1382 (25.83)	0.184
Obesity, n (%)	60 (10.89)	542 (10.13)	0.574
Obstructive sleep apnea, n (%)	25 (4.54)	298 (5.57)	0.311
GERD, n (%)	15 (2.72)	64 (1.2)	0.003
Chronic rhinitis, n (%)	8 (1.45)	5 (0.09)	<0.001
Atopic dermatitis, n (%)	12 (2.18)	6 (0.11)	<0.001
Anxiety, n (%)	16 (2.9)	166 (3.1)	0.798
Depression, n (%)	22 (3.99)	185 (3.46)	0.515
COPD, n (%)	24 (4.36)	439 (8.2)	0.001
Hypertension, n (%)	139 (25.23)	1723 (32.2)	0.001
Ischemic heart disease, n (%)	9 (1.63)	179 (3.35)	0.029
Atrial fibrillation, n (%)	42 (7.62)	603 (11.27)	0.009
Hypothyroidism, n (%)	41 (7.44)	284 (5.31)	0.037
Hyperthyroidism, n (%)	6 (1.09)	37 (0.69)	0.296
Diabetes mellitus, n (%)	69 (12.52)	914 (17.08)	0.006
Hypotension, n (%)	24 (4.36)	331 (6.19)	0.085
Syncope/collapse, n (%)	8 (1.45)	95 (1.78)	0.581
Nausea/vomiting, n (%)	7 (1.27)	40 (0.75)	0.189
Abdominal pain, n (%)	10 (1.81)	59 (1.1)	0.139
Acute respiratory failure, n (%)	94 (17.06)	529 (9.89)	<0.001
Urticaria, n (%)	18 (3.27)	115 (2.15)	0.092
Invasive mechanical ventilation, n (%)	84 (15.25)	593 (11.08)	0.004
Non-invasive mechanical ventilation, n (%)	22 (3.99)	132 (2.47)	0.032
Admission to ICU, n (%)	182 (33.03)	1699 (31.75)	0.539
IHM, n (%)	18 (3.27)	264 (4.93)	0.081
Severe anaphylaxis (IHM or/and admission to ICU), n (%)	189 (34.30)	1795 (33.55)	0.721

GERD: Gastroesophageal reflux disease; COPD: chronic obstructive pulmonary disease. ICU: Intensive care unit. IHM: In-hospital mortality.

**Table 3:** Characteristics, chronic conditions, specific signs, symptoms, and hospital outcomes of hospital admissions with a diagnosis of anaphylaxis in Spain, 2016–2021, according to the presence of asthma, after propensity score matching.

	Asthma	No Asthma	*p*
Age, mean (SD)	53.34 (19.02)	52.72 (18.32)	0.586
Women, n (%)	361 (65.52)	368 (66.79)	0.656
Smoking, n (%)	88 (15.97)	76 (13.79)	0.310
Anaphylactic reaction due to food, n (%)	132 (23.96)	128 (23.23)	0.777
Anaphylactic reaction due to serum, n (%)	6 (1.09)	6 (1.09)	1.000
Anaphylactic reaction due to drugs, n (%)	285 (51.72)	286 (51.91)	0.952
Anaphylactic shock, unspecified, n (%)	128 (23.23)	130 (23.59)	0.887
Obesity, n (%)	60 (10.89)	54 (9.8)	0.553
Obstructive sleep apnea, n (%)	25 (4.54)	23 (4.17)	0.768
GERD, n (%)	15 (2.72)	11 (2)	0.427
Chronic rhinitis, n (%)	8 (1.45)	5 (0.91)	0.403
Atopic dermatitis, n (%)	12 (2.18)	5 (0.91)	0.087
Anxiety, n (%)	16 (2.9)	13 (2.36)	0.572
Depression, n (%)	22 (3.99)	14 (2.54)	0.175
COPD, n (%)	24 (4.36)	23 (4.17)	0.881
Hypertension, n (%)	139 (25.23)	141 (25.59)	0.890
Ischemic heart disease, n (%)	9 (1.63)	6 (1.09)	0.435
Atrial fibrillation, n (%)	42 (7.62)	42 (7.62)	1.000
Hypothyroidism, n (%)	41 (7.44)	40 (7.26)	0.908
Hyperthyroidism, n (%)	6 (1.09)	3 (0.54)	0.315
Diabetes mellitus, n (%)	69 (12.52)	58 (10.53)	0.299
Hypotension, n (%)	24 (4.36)	28 (5.08)	0.570
Syncope/collapse, n (%)	8 (1.45)	10 (1.81)	0.635
Nausea/vomiting, n (%)	7 (1.27)	0 (0)	NA
Abdominal pain, n (%)	10 (1.81)	8 (1.45)	0.635
Acute respiratory failure, n (%)	94 (17.06)	47 (8.53)	<0.001
Urticaria, n (%)	18 (3.27)	17 (3.09)	0.864
Invasive mechanical ventilation, n (%)	84 (15.25)	49 (8.89)	0.001
Non-invasive mechanical ventilation, n (%)	22 (3.99)	11 (2)	0.052
Admission to ICU, n (%)	182 (33.03)	171 (31.03)	0.478
IHM, n (%)	18 (3.27)	9 (1.63)	0.079
Severe anaphylaxis (IHM or/and admission to ICU), n (%)	189 (34.3)	172 (31.22)	0.275

GERD: Gastroesophageal reflux disease; COPD: chronic obstructive pulmonary disease. ICU: Intensive care unit. IHM: In-hospital mortality. NA: not available due to small sample size.

**Table 4:** Multivariate analysis of the factors associated with severe anaphylaxis during hospital admission with a diagnosis of anaphylaxis in Spain, 2016–2021, according to asthma status.

		Asthma	All
		OR (95% CI)	OR (95% CI)
Age, years	18–44 years	1	1
	45–64 years	0.73 (0.36–1.48)	1.09 (0.9–1.32)
	≥65 years	1.28 (0.73–2.24)	1.32 (1.1–1.58)
Sex	Women	0.95 (0.58–1.54)	0.89 (0.65–1.23)
Ischemic heart disease	Yes	3.38 (1.33–9.84)	2.53 (1.84–3.47)
Acute respiratory failure	Yes	2.86 (1.6–5.11)	2.4 (1.55–3.72)
Invasive mechanical ventilation	Yes	3.85 (1.34–11.05)	4.21 (1.85–9.62)
Asthma	Yes	NA	0.84 (0.63–1.14)

OR: Odds ratio. CI: Confidence interval.

## Data Availability

According to the contract signed with the Spanish Ministry of Health and Social Services, which provided access to the databases from the Spanish National Hospital Database (Registro de Actividad de Atención Especializada. Conjunto Mínimo Básico de Datos, Registry of Specialized Health Care Activities. Minimum Basic Data Set), we cannot share the databases with any other investigator, and we have to destroy the databases once the investigation has concluded. Consequently, we cannot upload the databases to any public repository. However, any investigator can apply for access to the databases by filling out the questionnaire available at https://www.sanidad.gob.es/estadEstudios/estadisticas/estadisticas/estMinisterio/SolicitudCMBD.htm (accessed on 20 August 2023). All other relevant data are included in the paper.

## Supplementary Materials

The following supporting information can be downloaded at https://www.mdpi.com/article/10.3390/healthcare11233016/s1, Table S1: ICD-10 codes. Table S2: Characteristics, chronic conditions, specific signs, and symptoms of hospital admissions with and without a diagnosis of asthma in Spain, 2016–2021, according to the severity of anaphylaxis. Table S3: Multivariable analysis of the chronic conditions presented on admission and associated with severe anaphylaxis in Spain, 2016–2021, according to asthma status.
