# Supplementary material for: Trends in Anaphylaxis Hospitalizations among Adults in Spain and Their Relationship with Asthma—Analysis of Hospital Discharge data from 2016 to 2021
Source: Healthcare (Basel). 2023 Nov 22;11(23):3016. doi: 10.3390/healthcare11233016 (PMC10706569; doi:10.3390/healthcare11233016)
Supplement: Supplementary file 1 [file healthcare-11-03016-s001.zip › healthcare-2659129-supplementary.pdf]

Table S1. ICD-10 codes.

Variable	ICD-10
Anaphylaxis	T78.0xx, T78.2xxx, T80.5xxx, T88.6xxx
Anaphylactic reaction due to food, n (%)	T78.0xx
Anaphylactic reaction due to serum, n (%)	T80.5xxx
Anaphylactic reaction due to drugs, n (%)	T88.6xxx
Anaphylactic shock, unspecified, n (%)	T78.2xxx
Asthma, n (%)	J45
Obesity, n (%)	E66
Obstructive sleep apnea, n(%)	G47.3
GERD, n(%)	K21
Chronic rhinitis, n(%)	J31.0
Atopic dermatitis, n(%)	L20
Anxiety, n (%)	F41
Depression, n(%)	F32
COPD, n(%)	J44
Hypertension, n(%)	I10
Ischemic heart disease, n(%)	I21
Atrial fibrillation, n(%)	I48
Hypothyroidism, n(%)	E03
Hyperthyroidism, n(%)	E05
Diabetes mellitus, n(%)	E11
Hypotension, n(%)	I95
Syncope/collapse, n(%)	R55
Nausea/vomiting, n(%)	R11.2
Abdominal pain, n(%)	R10
Acute respiratory failure, n(%)	J96.0
Urticaria, n(%)	L50
Noninvasive mechanical ventilation, n (%)	5A09357, 5A09457, 5A09557
Invasive mechanical ventilation, n (%)	5A1945Z, 5A1955Z, 5A1935Z

GERD: Gastroesophageal reflux disease; COPD: chronic obstructive pulmonary disease

Table S2. Characteristics, chronic conditions, specific signs and symptoms of hospital admissions with and without a diagnosis of asthma in Spain, 2016-2021 according to the severity of anaphylaxis.

	**Asthma**			**No asthma**		
	**Severe**	**Not severe**	**p**	**Severe**	**Not severe**	**p**
N	189	362		1795	3556	
Age, mean (SD)	51.95(18.21)	54.06(19.41)	0.049	61.44(15.84)	60.69(18.05)	0.086
18-44 years, n (%)	62(32.8)	129(35.64)	0.047	259(14.43)	706(19.85)	0.075
45-64 years, n (%)	77(40.74)	112(30.94)		680(37.88)	1233(34.67)	
≥65 years, n (%)	50(26.46)	121(33.43)		856(47.69)	1617(45.47)	
Women, n (%)	113(59.79)	248(68.51)	0.041	831(46.3)	1775(49.92)	0.012
Smoking, n(%)	29(15.34)	59(16.3)	0.772	310(17.27)	504(14.17)	0.003
Anaphylactic reaction due to food, n (%)	47(24.87)	85(23.48)	0.717	152(8.47)	463(13.02)	<0.001
Anaphylactic reaction due to serum, n (%)	1(0.53)	5(1.38)	0.360	60(3.34)	97(2.73)	0.208
Anaphylactic reaction due to drugs, n (%)	98(51.85)	187(51.66)	0.965	1125(62.67)	2058(57.87)	0.001
Anaphylactic shock, unspecified, n (%)	43(22.75)	85(23.48)	0.847	448(24.96)	934(26.27)	0.302
Obesity, n (%)	20(10.58)	40(11.05)	0.867	207(11.53)	335(9.42)	0.016
Obstructive sleep apnea, n(%)	9(4.76)	16(4.42)	0.855	112(6.24)	186(5.23)	0.129
GERD, n(%)	4(2.12)	11(3.04)	0.528	19(1.06)	45(1.27)	0.511
Chronic rhinitis, n(%)	3(1.59)	5(1.38)	0.848	1(0.06)	4(0.11)	0.521
Atopic dermatitis, n(%)	4(2.12)	8(2.21)	0.943	4(0.22)	2(0.06)	0.086
Anxiety, n (%)	7(3.7)	9(2.49)	0.419	53(2.95)	113(3.18)	0.654
Depression, n(%)	6(3.17)	16(4.42)	0.478	65(3.62)	120(3.37)	0.641
COPD, n(%)	13(6.88)	11(3.04)	0.036	145(8.08)	294(8.27)	0.811
Hypertension, n(%)	53(28.04)	86(23.76)	0.272	603(33.59)	1120(31.5)	0.121
Ischemic heart disease, n(%)	8(4.23)	1(0.28)	0.001	94(5.24)	85(2.39)	<0.001
Atrial fibrillation, n(%)	13(6.88)	29(8.01)	0.634	262(14.6)	341(9.59)	<0.001
Hypothyroidism, n(%)	8(4.23)	33(9.12)	0.038	83(4.62)	201(5.65)	0.113
Hyperthyroidism, n(%)	1(0.53)	5(1.38)	0.360	12(0.67)	25(0.7)	0.886
Diabetes mellitus, n(%)	23(12.17)	46(12.71)	0.856	300(16.71)	614(17.27)	0.611
Hypotension, n(%)	6(3.17)	18(4.97)	0.326	117(6.52)	214(6.02)	0.473
Syncope/collapse, n(%)	1(0.53)	7(1.93)	0.191	21(1.17)	74(2.08)	0.017
Nausea/vomiting, n(%)	3(1.59)	4(1.1)	0.631	6(0.33)	34(0.96)	0.013
Abdominal pain, n(%)	4(2.12)	6(1.66)	0.702	11(0.61)	48(1.35)	0.015
Acute respiratory failure, n(%)	57(30.16)	37(10.22)	<0.001	317(17.66)	212(5.96)	<0.001
Urticaria, n(%)	7(3.7)	11(3.04)	0.677	22(1.23)	93(2.62)	0.001
Invasive mechanical ventilation, n (%)	75(39.68)	9(2.49)	<0.001	515(28.69)	78(2.19)	<0.001
Noninvasive mechanical ventilation, n (%)	13(6.88)	9(2.49)	0.012	94(5.24)	38(1.07)	<0.001

GERD: Gastroesophageal reflux disease; COPD: chronic obstructive pulmonary disease. ICU: Intensive care unit. IHM: In-hospital mortality.

Table S3. Multivariable analysis of the chronic conditions presents on admission and associated with severe anaphylaxis in Spain, 2016-2021 according to asthma status.

		**Asthma**	**All**
		**OR (95%CI)**	**OR (95%CI)**
Age, years	18-44 years	1	1
	45-64 years	0.81(0.46-1.45)	0.88(0.6-1.27)
	≥65 years	1.34(0.84-2.13)	1.21(1.01-1.44)
Sex	Women	0.89(0.59-1.89)	0.75(0.5-1.13)
Ischemic heart disease	Yes	4.59(1.48-14.29)	3.23(2.66-4.01)
Asthma	Yes	NA	0.89(0.68-1.19)
